# Soil phosphorus transformation and plant uptake driven by phosphate-solubilizing microorganisms

**DOI:** 10.3389/fmicb.2024.1383813

**Published:** 2024-03-27

**Authors:** Fei Pang, Qing Li, Manoj Kumar Solanki, Zhen Wang, Yong-Xiu Xing, Deng-Feng Dong

**Affiliations:** ^1^College of Agriculture, Guangxi University, Nanning, China; ^2^Guangxi Key Laboratory of Agricultural Resources Chemistry and Biotechnology, Smart Agricultural College, Yulin Normal University, Yulin, China; ^3^Department of Life Sciences and Biological Sciences, IES University, Bhopal, India

**Keywords:** phosphorus-solubilizing microorganisms, phosphorus, organic acids, phosphatase, arbuscular mycorrhizal fungi

## Abstract

Phosphorus (P) is an important nutrient for plants, and a lack of available P greatly limits plant growth and development. Phosphate-solubilizing microorganisms (PSMs) significantly enhance the ability of plants to absorb and utilize P, which is important for improving plant nutrient turnover and yield. This article summarizes and analyzes how PSMs promote the absorption and utilization of P nutrients by plants from four perspectives: the types and functions of PSMs, phosphate-solubilizing mechanisms, main functional genes, and the impact of complex inoculation of PSMs on plant P acquisition. This article reviews the physiological and molecular mechanisms of phosphorus solubilization and growth promotion by PSMs, with a focus on analyzing the impact of PSMs on soil microbial communities and its interaction with root exudates. In order to better understand the ability of PSMs and their role in soil P transformation and to provide prospects for research on PSMs promoting plant P absorption. PSMs mainly activate insoluble P through the secretion of organic acids, phosphatase production, and mycorrhizal symbiosis, mycorrhizal symbiosis indirectly activates P via carbon exchange. PSMs can secrete organic acids and produce phosphatase, which plays a crucial role in soil P cycling, and related genes are involved in regulating the P-solubilization ability. This article reviews the mechanisms by which microorganisms promote plant uptake of soil P, which is of great significance for a deeper understanding of PSM-mediated soil P cycling, plant P uptake and utilization, and for improving the efficiency of P utilization in agriculture.

## Introduction

Phosphorus (P) is an essential nutrient for plant growth and development, playing an important role in the synthesis of DNA, cell membrane components (phospholipids), adenosine triphosphate (ATP), respiration, and photosynthesis (Kafle et al., 2019; Bai et al., 2020). P in soil includes two forms: organic and inorganic P. Although soil contains a large amount of phosphorus, it usually exists in a form that cannot be directly utilized by plants (Ikhajiagbe et al., 2020; Divjot et al., 2021). P combines with Ca, Fe, and Al metals to form minerals, and P is adsorbed by iron/aluminum (hydrogen) oxides, leading to P fixation in the soil (Ma J. et al., 2021; Zhou J. et al., 2021). The mobility of P is poor in soil, and plants cannot directly absorb and utilize P, leading to the widespread phenomenon of low P in ecosystems, which limits plant growth and yield. A large amount of P fertilizer is applied during production to meet the P demand of plants. Because of the adsorption and fixation effects of soil on P, the applied P fertilizer rapidly becomes fixed, resulting in a P fertilizer utilization efficiency of only 10–25% (Dejene et al., 2023; Dong et al., 2023). Moreover, fixed P in the soil can lead to non-point source pollution, resulting in large amounts of P fertilizer flowing into water bodies, in turn leading to groundwater eutrophication, which is not conducive to the sustainable development of the ecological environment (Lyu et al., 2023; Wang et al., 2023). Phosphate ore is a nonrenewable resource. Half of the world’s existing P reserves are predicted to be depleted within 50–100 years (Zhu et al., 2018). Therefore, improving the utilization efficiency of P in soil is crucial for promoting plant growth, reducing environmental pollution, and improving resource management.

Phosphate-solubilizing microorganisms (PSMs) can convert soil P into a form that plants can absorb and utilize, and the application of PSMs is currently an important measure for increasing the available P content in soil (Yadav et al., 2017; Yadav, 2020). Many types of microorganisms dissolve P, which plays an important role in P cycling processes such as organic P mineralization, insoluble inorganic P dissolution, and P absorption (Zhu et al., 2018; Wise et al., 2021). Inoculating PSMs is an environmentally friendly method to promote crop productivity and understanding the mechanism of P solubilization by PSMs is of great significance for plants to adapt to low P stress and improve P utilization efficiency (Billah et al., 2019). This article discusses the types and functions of PSMs, P-solubilization mechanisms, main functional genes, and the impact of composite inoculation of PSMs on plant P partitioning, emphasizing the role of PSMs in plant P acquisition and utilization, and—based on this—proposes issues and corresponding measures that need to be considered in future research and applications of PSMs.

## Types and functions of PSMs

PSMs are widely distributed in nature, and microorganisms with P-solubilizing functions include bacteria, fungi, actinomycetes, and cyanobacteria, among which P-solubilizing fungi account for 0.1–0.5% of PSMs, and P-solubilizing bacteria account for 1–50% of the total (Fatima et al., 2022). P-solubilizing bacteria included 34 genera, including *Bacillus*, *Pseudomonas*, *Escherichia*, and *Burkholderia*. Of these, *Bacillus*, *Pseudomonas*, and *Acinetobacter* have been studied extensively (Divjot et al., 2021; Timofeeva et al., 2022). P-solubilizing fungi include *Arbuscularmy* sp., *Aspergillus*, *Penicillium*, among which *Aspergillus* is the most reported, followed by *Penicillium* (Jiang et al., 2020; Divjot et al., 2021; Etesami et al., 2021). P-solubilizing fungi produce 10 times more organic acids than P-solubilizing bacteria and can increase the contact area with the soil through the mycelium, thereby increasing the application potential of P-solubilizing fungi (Jiang et al., 2020). The main P-solubilizing actinomycetes are *Streptomyces* and *Micromonospora* (Aallam et al., 2021; De Zutter et al., 2022). Microorganisms not only promote the conversion of difficult-to-utilize P to available P but also assist plants with absorbing P outside the rhizosphere, thus playing an important role in the process of plant P acquisition (Castagno et al., 2021).

PSMs not only have P-solubilizing effects but can also produce organic acids and iron carriers, regulate plant hormone levels, and fix nitrogen to promote the acquisition and growth of rice nutrients (Ribeiro et al., 2018; Unnikrishnan and Binitha, 2024). PSMs can secrete plant hormones such as auxins, cytokinins, and gibberellins, produce antifungal compounds and volatile bactericidal metabolites, and synthesize 1-aminocyclopropane-1-carboxylate (ACC) deaminase to improve phosphorus absorption and disease resistance, thereby increasing plant growth and yield (Hakim et al., 2021; Rawat et al., 2021). PSMs can also secrete antibiotics, iron carriers, and lyases to protect plants from various soil-borne pathogens and promote plant growth (Toscano-Verduzco et al., 2020; Kumawat et al., 2021). Moreover, inoculation with PSMs can significantly affect the diversity and abundance of soil microbial communities and enhance the interactions between microorganisms, ultimately resulting in improved organic matter degradation and soil nutrient quality (Zhang X. et al., 2021). PSMs also have different functions in different ecological environments and can enhance crop resistance to certain abiotic stresses, including cold, salt, heavy metals, and drought (Table 1). The P-solubilizing bacterium *Bacillus atrophaeus* GQJK17 S8 can tolerate 11% NaCl, which can improve the germination rate, seedling biomass, and growth vitality index of quinoa plants (Mahdi et al., 2021). In addition, there are strains with different abiotic stress tolerance abilities, Such as *Pseudomonas* PGERs17, which is resistant to cold stress (Rizvi et al., 2021), *Bacillus* YMX5, which is resistant to high salt stress (Jiang et al., 2020), and *Streptomyces laurentii* EU-LWT3-69, which is resistant to drought stress (Toscano-Verduzco et al., 2020). This type of PSMs not only promotes plant P absorption but also helps plants grow in extreme environments.

**Table 1:** The effect of PSMs in abiotic stress, the application of PSMs to improve crop performance.

Phosphate-solubilizing microorganisms	Plant	Abiotic stress	Effect	Reference
*Lysinibacillus fusiformis* YJ4 *Lysinibacillus sphaericus* YJ5	Maize	Cold	Increased the lignification, osmolytes, phenolic content, phytohormones, the enzymatic antioxidant defenses and mineral contents	Jha and Mohamed (2023)
*Pseudomonas* sp. CIBEA71 *Pseudomonas* sp. CIBEB51	Wheat	Cold	Produce phosphorus-solubilization halos and increase root length	Yarzábal et al. (2018)
*Acinetobacter rhizosphaerae* EU-KL44	Wheat	Cold	Increase the shoot length and root length, and improve plant physiological and growth parameters	Kour and Yadav (2023)
*Pseudomonas* sp. GBPI_506 *Pseudomonas palleroniana* GBPI_508 *Pseudomonas proteolytica* GBPI_Hb61 *Pseudomonas azotoformans* GBPI_CDB143	*Arabidopsis thaliana*	Cold	Promoted plant rosette diameter, leaf area, and biomass growth	Adhikari et al. (2021)
*Bacillus subtilis* TPB4 *Bacillus halotolerans* TPB19 *Bacillus pumilus* TPB30	Cotton	Heat	Increased seedling growth and improve cotton yield and biomass	Shah et al. (2022)
*Streptomyces laurentii* EU-LWT3-6 *Penicillium* sp. EU-DSF-10	Millet	Drought	Increased plant chlorophyll content, and decreased lipid peroxidation	Kour et al. (2020)
*Pseudomonas helmanticensis* B30 *Pseudomonas baetica* B21	Wheat	Drought	Increase wheat growth indices, grain yield, and shoot phosphorus uptake	Karimzadeh et al. (2021)
*Enterobacter ludwigii* SH-6	Maize	Drought	Improve seeds germination performance and increase seedling drought tolerance	Shaffique et al. (2022)
*Microbacterium* sp., *Streptomyces* sp.	*Quercus brantii*	Drought	Increase root length and weight, and enhance growth and physiological traits of seedlings	Zolfaghari et al. (2021)
*Bacillus* Y8	Sugarcane	Drought	Enhanced plant biomass and root length	Wang et al. (2020)
*Paenibacillus polymyxa* IA7 *Bacillus subtilis* IA6	Cotton	Drought	Improve seedlings growth and change root architecture	Ahmad et al. (2021)
*Pseudomonas azotoformans* N76	Wheat	Salt	Increase seed germination percentage, shoot and root length, fresh and dry weights	Belkebla et al. (2022)
*Penicillium funicuiosum* P1	Quinoa	Saline-alkali stress	Promoted the antioxidant system and photosynthesis	Jin et al. (2022)
*Bacillus cereus* WGT1 *Bacillus thuringiensis* WGT11	Wheat	Salt	Produce plant growth promoting substances and enhance wheat grain yield	Aliyat et al. (2022)
*Kocuria rhizophila* Y1	Maize	Salt	Improved plant growth performance, biomass production, seed germination rate, and antioxidant levels	Li et al. (2020)
*Bacillus megaterium* PSB1 *Staphylococcus haemolyticus* PSB2 *Bacillus licheniformis* PSB3	Mung beans	Heavy metals	Increased seeds germination rate and growth	Biswas et al. (2018)
*Bacillus atrophaeus* GQJK17 S8	Quinoa	Heavy metals and salt	Enhanced seedling growth and biomass, and improved the germination rate	Mahdi et al. (2021)
*Burkholderia* sp. N3	Watermelon	Heavy metal	Restore bacterial structure and improve the total dry weight	Zhang J. et al. (2022)

## The P removal mechanism of PSMs

**Figure 1:**
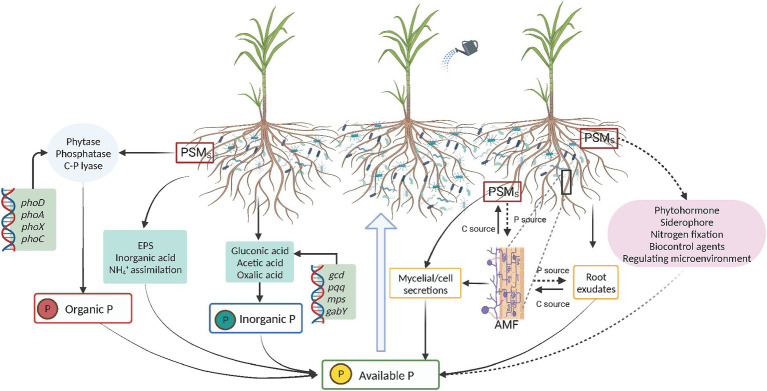
PSMs promote the production of available phosphorus and plant absorption through multiple pathways. AMF, arbuscular mycorrhizal fungi; PSMs, phosphate solubilizing microorganisms; EPS, extracellular polysaccharides.

P in soil includes two forms: inorganic and organic P. Inorganic P usually exists as phosphates, divided into soluble and insoluble P. Insoluble P mainly includes phosphates such as aluminum phosphate, iron phosphate, magnesium phosphate, and calcium phosphate (Aliyat et al., 2022), while soluble P mainly exists as hydrogen phosphate and dihydrogen phosphate ions (HPO_4_^2−^ and H_2_PO_4_^−^) (Hao et al., 2020; Divjot et al., 2021; Li et al., 2021). Organic P mainly includes P-containing organic compounds, such as orthophosphate monoesters, orthophosphate diesters, organic polyphosphates, and phosphonates (Li C. et al., 2019). Organic acids and phosphatases produced by microorganisms are crucial for the cycling of inorganic and organic phosphorus in soil (Rasul et al., 2021). Insoluble inorganic P is mainly dissolved by organic acids, and enzymatic hydrolysis is the main method used to dissolve the organic forms of P (Figure 1). The mechanisms of microbial P solubilization can be divided into the following types:

### Secretion of organic acids

Organic acids secreted by PSMs transform insoluble inorganic P into plant-usable P. Microorganisms produce organic acids in two ways: physiological secretion and decomposition of organic matter (Schneider et al., 2019). The organic acids secreted by PSMs include gluconic, lactic, citric, and oxalic acids (Kalayu, 2019; Azaroual et al., 2020). Organic acids mainly dissolve insoluble inorganic P as follows: organic acid anions compete with phosphate ions for binding sites on soil particles, reducing soil adsorption of phosphate ions; complex metal ions such as iron, aluminum, and calcium in soil to release bound phosphate ions; and reduction of the pH of the medium promotes the dissolution of insoluble inorganic P (Adeleke et al., 2017; Rawat et al., 2021). The organic acids secreted by PSMs not only enhance the solubility of insoluble P, such as apatite and calcium phosphate, but also chelate with cations such as Ca^2+^, Fe^3+^, Al^3+^, and Mg^2+^, and organic acid anions compete with inorganic P physically or electrostatically for the same adsorption sites in the soil, releasing phosphate ions and increasing the effective P content (Rawat et al., 2021). The organic acids produced by microorganisms chelate with cations through hydroxyl and carboxyl groups, transforming phosphates into soluble forms and increasing the effective P content (Bhattacharyya et al., 2016). Many bacteria secrete organic acids (carboxylic acids) that can increase the solubility of calcium phosphate (Jayakumar et al., 2019). In addition, organic acids can promote the dissolution of insoluble inorganic phosphate compounds, such as tricalcium phosphate, dicalcium phosphate, hydroxyapatite, and phosphate rock, thereby improving the utilization rate of phosphate fertilizers (Oteino et al., 2015; Cheng et al., 2017). PSMs secrete various organic acids to convert insoluble inorganic phosphorus into soluble orthophosphates that are easily absorbed by plants (Venkiteshwaran et al., 2021; Campos et al., 2023). The types, contents, and phosphate solubility of organic acids produced by PSM vary, such as of *Enterobacter* sp. strain 15S, can produce organic acids such as citric, fumaric, ketoglutaric, malic, and oxalic acids (Zuluaga et al., 2023). However, *Trichoderma* sp. produce different types of organic acids, including lactic acid, fuzzy acid, ascorbic acid, isocitric acid, malic acid, citric acid, and phytic acid (Bononi et al., 2020).

Gluconic acid is considered a common and important organic acid, and is one of the many organic acids produced by microorganisms that have been extensively studied (Kaur et al., 2021). Glucose can form gluconic acid through the synergistic effects of pyrroloquinoline quinine (PQQ) and glucose dehydrogenase (GDH), which dissolves insoluble phosphate (Jaiswal et al., 2021). *Pseudomonas* produces gluconic acid to increase phosphate solubility, which has become an important technology for improving phosphate fertilizer management in modern agriculture (Wang et al., 2022; Rai et al., 2023). Inoculation of *Pseudomonas fluorescens* and *Pseudomonas putida* under soluble phosphate-restricted conditions can produce a large amount of gluconic acid, which promotes plant growth (Jin et al., 2022).

### Enzymatic hydrolysis

Organic P cannot be directly absorbed by plants but needs to be mineralized into inorganic P before it can be utilized by plants. Enzymatic hydrolysis is the main way to mineralize organic P under conditions of low available P content, PSMs can hydrolyze organic P through biological enzymes, such as phosphatase, phytase, and C-P lyase (Stefanoni Rubio et al., 2016; Prabhu et al., 2019). Two hydrolytic enzymes, phytase and phosphatase, play important roles in PSM mineralization (Liu et al., 2022). Phytase is an extracellular enzyme involved in the mineralization process of soil P, and phytase produced by microorganisms can release orthophosphate from phytate organic compounds, converting P into a form that can be utilized by plants (Ortega-Torres et al., 2021; Timofeeva et al., 2022). Microbial phytase activity is closely related to its ability to dissolve phosphorus (Ben Zineb et al., 2020).

PSMs not only secrete phytase, but also produce phosphatase to mineralize organic P. Phosphatases are divided into acid phosphatase (ACP) and alkaline phosphatase (ALP), and their existence is greatly influenced by the acidity and alkalinity of the environment. ACP is more abundant in acidic soils, while ALP dominates in neutral and alkaline soils (Borges et al., 2021; Cheng et al., 2023). The activity of ALP is inhibited by inorganic phosphates in the environment, while ACP activity is not inhibited by high levels of phosphates (Li et al., 2021; Xie et al., 2021). In addition, temperature can affect phosphatase activity, and an increase in temperature can enhance the activity of phosphatases secreted by PSMs (Hessen et al., 2017; Jiang et al., 2018). Phosphatases are responsible for mineralizing approximately 90% of the organic P in soils, except phytates (Alori et al., 2017; Chen and Arai, 2023). Many microorganisms, including *Aspergillus*, *Bacillus,* and *Pseudomonas*, produce phosphatases (Shrivastava et al., 2018; Kaur and Chatli, 2019; Zaborowska et al., 2020). Purified ALP from *Bacillus licheniformis* MTCC 2312 has been added to sterilized soil, which improved the phosphate content in the roots and stems of maize (Singh and Banik, 2019).

### The role of mycorrhizal symbiosis

**Figure 2:**
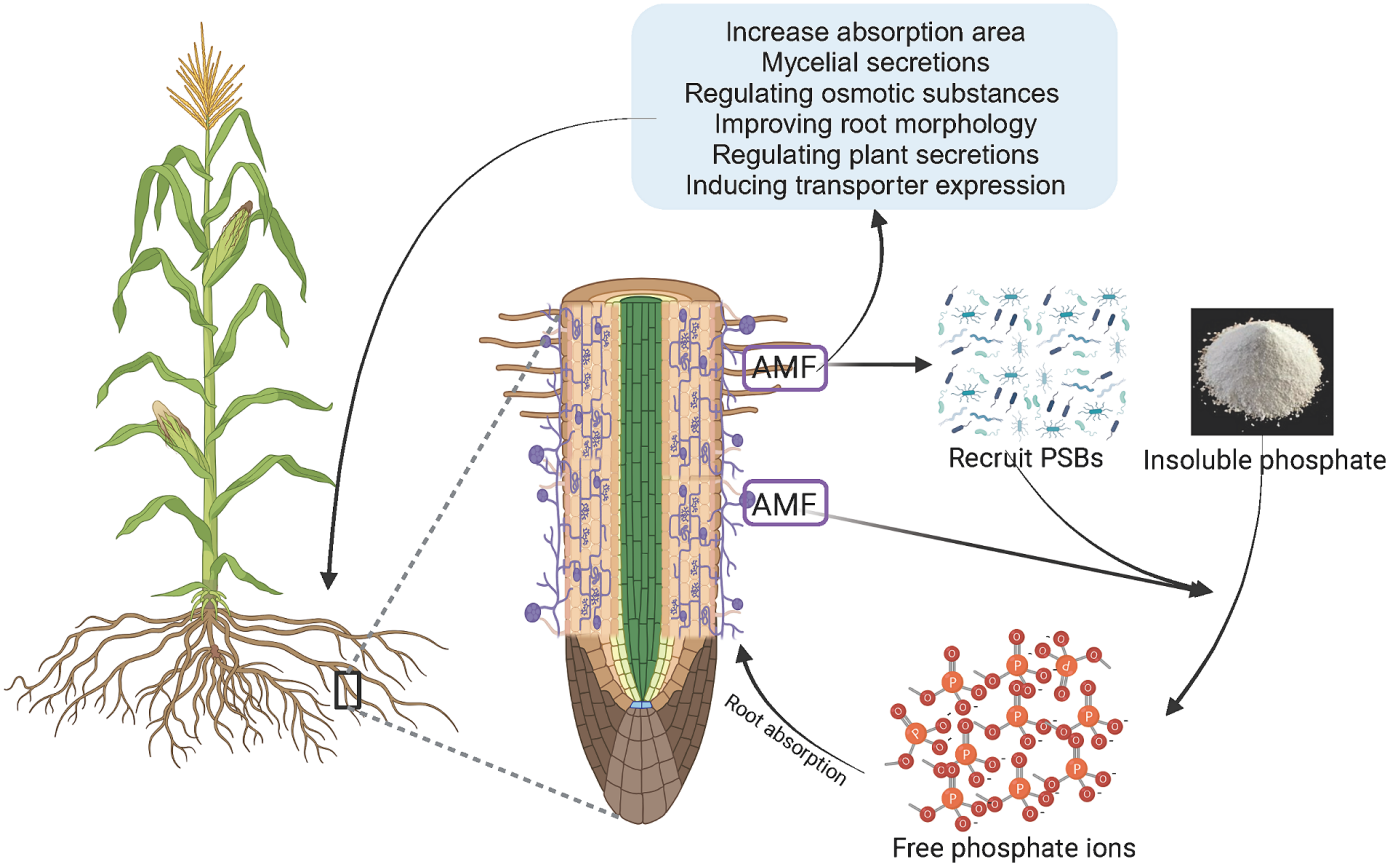
AMF recruiting PSBs or directly activating phosphorus elements to enhance plant phosphorus absorption. AMF, arbuscular mycorrhizal fungi; PSB, phosphate solubilizing bacteria.

Mycorrhizal fungi can form mutualistic symbioses with plant roots and help plants absorb mineral elements and water from the soil, while plants provide carbohydrates to the fungi (Chiu and Paszkowski, 2019; Genre et al., 2020). Arbuscular mycorrhizal fungi (AMF) can form mycorrhizal fungi in symbiosis with 70–80% of terrestrial plants, which is an effective way for plants to obtain P (Shi et al., 2021). The symbiotic interface between AMF and plants is an important site for material exchange between plants and fungi, which can increase the range of plant P absorption and the transport of P to root cells (Bao et al., 2022). Plants absorb P through a series of morphological changes to expand the surface area of the roots and improve the exchange interface between the roots and soil when subjected to low P stress. Examples of this include increasing the number and length of root hairs; increasing the root-to-shoot ratio; adjusting the angle of root growth; increasing the number of lateral roots, adventitious roots, and young roots; increasing root length, shallow roots; and increasing root length and density in the soil surface layer (Hammelehle et al., 2018; Lynch, 2019; Zhang Z. et al., 2021). Mycorrhizal plants can obtain P from the soil through the root pathway absorbed by root hairs and root epidermal cells, as well as the hyphal pathway absorbed by arbuscular mycorrhizal fungi, which synergistically promote nutrient absorption (Ferrol et al., 2019; Chu et al., 2020; Zhou J. et al., 2021). In the hyphal pathway, arbuscular mycorrhizal fungi improve plant P nutrient status through the hyphae. Hyphae not only penetrate soil pores smaller than root hairs but also extend further from the root surface to obtain a larger range of P in the soil (Ma X. et al., 2021; Gregory, 2022). While expanding the absorption range, AMF can stimulate the secretion of organic acids and ACP by host plant roots; their own mycelia can also secrete organic acids and ACP, reduce the pH of the surrounding soil, and convert insoluble phosphates into available P, which has the similar function as phosphate-solubilizing bacteria (Zhang L. et al., 2022; Xing et al., 2023). Organic acids, carbohydrates, amino acids, plant hormones, and other substances have been found in the mycelial secretions of AMF (*Rhizophagus clarius* and *Rhizophagus irregularis*) (Luthfiana et al., 2021). Plants are likely to form mycorrhizal symbioses with mycorrhizal fungi to enhance their ability to obtain P under P-deficient conditions (Raven et al., 2018). The absorption of soil P through mycorrhizal fungi is an effective way for plants to supplement P (Figure 2).

### Other functions

Compared with organic acids, the efficiency of inorganic acids (such as sulfuric acid and nitric acid) produced by PSMs in dissolving phosphates is lower. *Nitrobacter* and *Thiobacillus* spp. produce inorganic acids, such as nitric acid and sulfuric acid, to dissolve P and increase the available P content in the soil (Shrivastava et al., 2018; Dipta et al., 2019). PSMs also produce extracellular polysaccharides (EPS) that can form complexes with metal ions and enhance the solubilization of P (Naseem et al., 2018; Thampi et al., 2023). The combined action of EPS and organic acids can dissolve Ca_3_(PO_4_)_2_, which adds EPS to the culture medium and increases the solubilization of tricalcium phosphate by organic acids (Mendoza-Arroyo et al., 2020; Liu et al., 2024). Ammonium (NH^4+^) present in soil is absorbed by PSMs to synthesize amino acids. Proton efflux caused by ammonium ion assimilation is another P-solubilization mechanism in microorganisms. *Bacillus marisflavi* FA7 is accompanied by ammonium ion assimilation, which decreases the pH of the culture medium and dissolves insoluble phosphates (Prabhu et al., 2018). PSMs can also promote P absorption by increasing root weight, root length, projection area, surface area, tip, and branch number (Liu X. et al., 2019; Galindo et al., 2022).

## Main P cycling functional genes of PSMs

With the continuous development of molecular biology, researchers have explored the mechanism of P solubilization from a genetic perspective. The specific molecular genetic mechanisms underlying mineral phosphate dissolution have not been clearly elucidated to date (Timofeeva et al., 2022). Research on the functional genes of PSMs has mainly focused on genes related to the microbial secretion of organic acids and phosphatase production.

### Organic acid-related genes

Gluconic acid is the main organic acid secreted by PSMs (Zhang et al., 2023). Genes related to gluconic acid synthesis are key for the regulation of P-solubilization ability. Gluconic acid is synthesized by the oxidation of glucose by GDH, which requires pyroquinoline quinone (PQQ) as a cofactor to participate in the reaction (Karagoz et al., 2020; Wu et al., 2022). PQQ synthesis involves six core genes (*pqqA*, *pqqB*, *pqqC*, *pqqD*, *pqqE*, and *pqqF*) associated with dehydrogenase activity and mineral phosphate dissolution in microorganisms (Wan et al., 2020; Dudeja et al., 2021; Joshi et al., 2023). The *pqqA* gene plays an important role in PQQ biosynthesis and P solubilization. Mutations in *pqqA* in *Rahnella aquatilis* HX2H significantly reduce the gluconic acid content in the culture medium, leading to a significant decrease in the solubility of mineral phosphates (Li et al., 2014). In addition, *pqqE* is highly conserved and crucial for the biosynthesis of PQQ (Ludueña et al., 2017; Lo et al., 2023). *Pantoea* sp. and *Pseudomonas* sp. carrying *pqqE* can solubilize P and increase crop yields (Tahir et al., 2020). The expression level of the *pqq* gene in *Serratia* sp. S119 increases under P-deficient growth conditions, catalyzing the oxidation of glucose to gluconic acid and alleviating P deficiency (Ludueña et al., 2017).

The membrane-bound quinoprotein glucose dehydrogenase (PQQGDH) is an important enzyme that regulates the synthesis of gluconic acid and dissolution of insoluble phosphate and is encoded by the *gcd* gene (Jha et al., 2019; Wu et al., 2022). The genes related to gluconic acid production include *gabY* and *mps* (Zhao et al., 2014; Rawat et al., 2021). *Pseudomonas* sp. MS16 was isolated from the rhizosphere soil of wheat and its P-solubilization activity was further validated through amplification, sequencing, and phylogenetic analysis of *gcd* gene (Suleman et al., 2018). The abundances of *gcd* genes were significantly correlated with environmental factors such as dissolved oxygen, phosphorus hydrochloride, and dissolved total phosphorus (Li Y. et al., 2019). The *gcd* gene can serve as a genetic marker to evaluate the potential of microorganisms to dissolve inorganic phosphorus. *Acinetobacter* sp. MR5 and *Pseudomonas* sp. MR7 carrying the *gcd* gene have the effect of promoting plant P absorption and growth, and rice plants treated with bacteria exhibited an increase in P content and grain yield of approximately 67 and 55%, respectively, compared with control plants (Rasul et al., 2019). However, the expression of *gcd* is inhibited by an increase in the soluble phosphate concentration (Zeng et al., 2016).

### Phosphatase genes

Phosphatases are important enzymes for mineralizing organic P and include ACP and ALP. ACP is mainly secreted by plants and fungi, whereas ALP is mainly produced by bacteria (Fraser et al., 2017). ALP and gluconic acid are important factors that affect the availability of P in soil (Liang et al., 2020; Wang et al., 2021). Among the enzymes involved in organic P mineralization, bacterial ALP has been extensively studied in terms of its biosynthesis, genetic control, and catalytic properties (Drozd et al., 2011; Kageyama et al., 2011; Sebastián and Ammerman, 2011; Park et al., 2022; Wijeratne et al., 2022). ALP is primarily encoded by *PhoA*, *PhoD*, and *PhoX* (Liu et al., 2018; Wang et al., 2021; Zhou Y. et al., 2021). PhoA hydrolyzes phosphate monoesters, whereas phoD and phoX decompose phosphate monoesters and phosphate diesters (Chen et al., 2019; Srivastava et al., 2021; Yuan et al., 2023). Among the genes encoding ALP, *phoD* is a key gene in soil microorganisms (Tan et al., 2013; Sun et al., 2019; Huang et al., 2020). The abundance of *phoD* in soil correlated positively with ALP activity and the available P concentration (Fraser et al., 2015; Wang et al., 2021; Xu et al., 2022). The *phoD* gene is used as a marker gene to estimate the abundance and community composition of organic P-mineralization microorganisms, thereby allowing investigation of the microbial regulatory mechanisms of phosphorus cycling (Hu et al., 2020; Azene et al., 2023). In addition to the ALP genes, the ACP genes mainly include *phoC*, whereas the phytase genes include *phyA, appA,* etc. The *phoC* gene is an important gene encoding acid phosphatase (Apel et al., 2007; Fraser et al., 2017). In neutral or low-pH soils, the *phoC g*ene is more dominant than the *phoD* gene (Fraser et al., 2017). After genetic transformation of maize using the phytase gene (*phyA2*) of *Aspergillus ficuum*, the growth and ability to obtain P from phytates were significantly improved (Jiao et al., 2021). In addition, a large number of studies have reported the isolation of various genes with P-solubilization ability from different species, such as *mMDH* from *Penicillium oxalicum* C2 (Lü et al., 2012), *vgb* from *Vitreoscilla hemoglobin* (Yadav et al., 2014), *Eno* from *Burkholderia cenococcia* 71-2 (Liu C. et al., 2019), *Zymomonas mobility* (*invB*), and *Saccharomyces cerevisiae* (*suc2*) (Kumar et al., 2016).

The use of metagenomic methods to analyze soil microbial P-cycling functional genes lays a solid foundation for the study of P-cycling genes, which helps us explore the potential functions of PSMs from a more comprehensive perspective. The P-cycling functional genes are mainly divided into three categories and seven functional groups, including those involved in P activation (including phosphate ester mineralization and inorganic phosphate dissolution), P absorption (phosphate ester transport and inorganic phosphate transport), and regulation of P-deficiency-induced responses (Liu et al., 2018; Dai et al., 2020; Siles et al., 2022). With the development of omics technologies and improvement of functional gene reference databases, researchers will more comprehensively reveal the functions and molecular mechanisms of microorganisms involved in plant soil P cycling.

## The effect of co-inoculation of PSMs on plant P acquisition

P-solubilizing bacteria and fungi on their own have a limited ability to mineralize organic P and solubilize inorganic P. Co-inoculation of plants with two or more strains promotes P absorption and plant growth (Table 2). The interaction between AMF and phosphate-solubilizing bacteria is more effective than inoculation alone for promoting P absorption and plant growth (El Maaloum et al., 2020; Wahid et al., 2020). The combination of bacteria and fungi has a synergistic effect, and mixed inoculation of AMF and P-solubilizing bacteria increases the root dry weight of plants by up to 58% compared with a single inoculation (Sharma et al., 2020). Mixed inoculation of AMF and PSMs not only improves soil fertility but also significantly increases crop yield. Inoculation with *Azospirillum brasilense* and *Bacillus subtilis* can improve the efficiency of P fertilizer utilization in sugarcane and positively affect the quality and yield of sugarcane crops (Rosa et al., 2020). Co-inoculation with *Trichoderma viride*, *Humicola* spp., *Paecilomyces lilacinus*, *Gluconacetobater diazotropicus*, *Azospiriillum brasilense*, and *Bacillus subtilis* can improve nutrient cycling and soil fertility, thereby promoting sugarcane root development (Tayade et al., 2019). Co-inoculation with phosphate-solubilizing bacteria and AMF significantly increases the soil enzyme activity and rhizosphere microbial count, and both synergistically promote nitrogen and P nutrient uptake (Varinderpal-Singh et al., 2020; Cozzolino et al., 2021). The composite inoculation of *Bradyrhizobium japonicum* 5038 and *Paenibacillus mucilaginosus* 3016 on soybeans resulted in a significant increase in the abundance of phosphorus cycle genes, as well as an increase in soil available phosphorus and phosphatase activity (Xing et al., 2022).

**Table 2:** The positive effect of co-inoculation PSMs on the plant growth and yield.

Phosphate-solubilizing microorganisms	Host	Growth condition	Impact on plant performance	Reference
*Rhizoglomus irregulare* QS69 *Pseudomonas fluorescens* PSB1 *Pseudomonas koreensis* PSB11 *Pseudomonas fluorescens* PSB18	*Solanum lycopersicum*	Pot	Increased the shoot dry weight and the root dry weight	Sharma et al. (2020)
*Rhizophagus irregularis* *Rahnella aquatilis* HX2	*Medicago truncatula*	Pot	Promoted the C-P exchange between plant and arbuscular mycorrhizal fungi	Duan et al. (2023)
*Suillus grevillea* *Cedecea lapagei*	*Pinus massoniana*	Pot	Increase soluble phosphorus content and promote phytate uptake	Mei et al. (2024)
*Azospirillum brasilens* Ab-V5 *Azospirillum brasilens* Ab-V6 *Bacillus subtilis* CCTB04 *Pseudomonas fluorescens* CCTB03	Sugarcane	Field	Improved dry matter, total phosphorus accumulation and stalk production, and reduced phosphorus fertilization	Rosa et al. (2020)
*Azospirillum brasilense* *Bacillus subtilis* *Pseudomonas fluorescens*	Sugarcane	Farm	Increased stalk yield and sugar, and reduced phosphate fertilization	Fernandes et al. (2023)
*Rhizophagus irregularis* *Talaromyces flavus* *Talaromyces helicus* L7B *Talaromyces helicus* N24 *Talaromyces diversus*	Wheat	Pot	Enhanced soil alkaline phosphatase activity and increased the symbiotic efficiency	Della Mónica et al. (2020)
*Rhizobium* sp. LSMR-32 *Enterococcus mundtii* LSMRS-3	Mungbean	Farm	Improved seed germination, plant height, biomass, chlorophyll content	Kumawat et al. (2021)
*Burkholderia vietnamiensis* KKUT8-1 *Rhizophagus aggregatus*	Sunchoke	Greenhouse	Increased plant water status, reduced electrolyte leakage, and reduced malondialdehyde and proline concentration	Nacoon et al. (2022)
*Bacillus licheniformis* PSB1 *Pantoea dispersa* PSB2 *Staphylococcus* sp. PSB3	Rice	Field	Curtailed phosphorus fertilizer dose and increased grain yield	Rawat et al. (2022)
*Funneliformis mosseae* *Bacillus megaterium* 10011	Alfalfa	Pot	Promoted mycorrhiza growth and the plant production performance	Liu et al. (2020)
*Bradyrhizobium japonicum* 5038 *Bacillus aryabhattai* MB35-5 *Paenibacillus mucilaginosus* 3016	Soybean	Pot	Increased the phosphorus metabolism-related genes abundance, phosphatase activities, the phosphorus content and soybean biomass.	Xing et al. (2022)
*Enterobacter asburiae* BFD160 *Pseudomonas koreensis* TFD26 *Pseudomonas linii* BFS112	Barattiere	Pot	Improve fruit yield, maturity, chlorophyll content, photosynthetic capacity, and gas exchange	Murgese et al. (2020)
*Azospirillum brasilens* Ab-V5 *Azospirillum brasilens* Ab-V6 *Bacillus subtilis* CCTB04 *Pseudomonas fluorescens* CCTB03	Sugarcane	Farm	Increased leaf phosphorus concentration and sugar yield	Rosa et al. (2022)
*Funneliformis mosseae* *Apophysomyces spartima*	Palm	Pot	Increased nutrient uptake and improved the gas-exchange and root growth	Zai et al. (2021)
*Bacillus megatherium* Compost tea	Sugar beet	Field	Enhanced the antioxidant system	Osman et al. (2022)
*Enterobacter ludwigii* AFFR02 *Bacillus megaterium* Mj1212	Alfalfa	Pot	Increase total phenolic content, total flavonoid, and superoxide dismutase	Kang et al. (2021)

The interactions between microorganisms to form complexes help plants absorb P. Fructose secreted by AMF can stimulate the expression of PSMs phosphatase genes, promote phosphatase synthesis and secretion, and increase the mineralization of organic P (Zhang et al., 2018). The mycelia of AMF can secrete compounds such as sugars, carboxylates, and amino acids, which can be utilized by phosphate-solubilizing bacteria (Cartabia et al., 2021; Weng et al., 2022). The exudate of AM fungi can serve as a source of carbon for bacteria as well as a signal and effector molecule that stimulates bacterial growth and activity (Zhang et al., 2016; Zeng et al., 2018; Zhang et al., 2018). In addition, bacteria on the surface of the mycelium can move along the mycelium and migrate to nutrient patches to activate organic P, thereby improving the utilization efficiency of the P in the soil by plants (Jiang et al., 2021). Bacteria colonize the surface of AMF mycelia to facilitate the acquisition of mycelial exudates (Emmett et al., 2021). Cooperation between AMF and bacteria is a manifestation of the symbiotic relationship between AMF and plants.

The symbiotic relationship between AMF and terrestrial plants is one of the most representative examples of microbial–plant cooperation (Brundrett and Tedersoo, 2018). The interaction between AMF and phosphate-solubilizing bacteria can affect the P exchange between plants and mycorrhizal fungi because the interaction between AMF and phosphate-solubilizing bacteria increases the secretion of phosphatase and gluconic acid, promoting the absorption and transport of P by extraradicular hyphae. P is transferred to plant roots by AMF, and plants, in turn, provide carbon sources to AMF, improving C-P exchange between plants and AMF (Duan et al., 2023). Under natural conditions, close cooperation between microorganisms is scientifically more effective for completing ecological functions than the independent actions of a single microorganism. *Suillus grevillea* synergistically mineralizes phytic acid by recruiting *Cedecea lapeti* and stimulates the upregulation of its own P-solubilization-related gene expression and growth of *Cedecea lapeti*, thereby promoting plant uptake of organic P (Mei et al., 2024). Ectomycorrhizal fungi recruit specific bacterial colonies by providing carbon sources, such as trehalose, mannitol, and organic acids (Deveau et al., 2010; Haq et al., 2017). These recruited bacteria can perform various ecological functions, such as promoting mycelial growth and assisting ectomycorrhizal fungi in absorbing nutrients (Tarkka et al., 2018; Pent et al., 2020). The co-inoculation of phosphate-solubilizing bacteria and fungi has shown good results in promoting plant growth, nutrient absorption, mycorrhizal symbiosis, and microbial biomass. In addition, the co-inoculation of PSMs with other functional microorganisms can achieve various goals to promote plant growth. For example, the co-inoculation of PSMs with nitrogen-fixing bacteria can increase the utilization of phosphorus and the fixation of nitrogen in the atmosphere, thereby improving soil fertility and crop yield, and promoting the development of sustainable agriculture (Zveushe et al., 2023). The co-inoculation of PSMs with biocontrol bacteria not only improves plant absorption of phosphorus, but also significantly reduces the incidence and severity of diseases, which is more effective than the single inoculation of PSMs (Nepomuceno et al., 2019).

## Conclusion and perspective

The low available P content in the soil and the low efficiency of P fertilizer utilization limit plant growth and yield. PSMs increase the soil available P content, improve the P fertilizer utilization efficiency, and promote plant growth. Therefore, the use of biological pathways to improve the utilization efficiency of P in soil has attracted the attention of scientists in various countries. Domestic and foreign scholars have identified many microorganisms with P-solubilization abilities through screening and conducted research on them. However, owing to factors such as the microbial P-solubilization ability, the colonization ability in the plant rhizosphere, and stability, relatively few strains have been applied in production practices to date. Many studies have focused on the effects of PSMs on plant growth under conventional cultivation conditions; however, little attention has been paid to their effects on plant growth under abiotic stress conditions. With the continuous development of molecular technologies and genomics, related P-solubilizing genes are constantly being explored; however, gene research mainly focuses on functional verification, and the interaction mechanism between P-solubilizing genes is still unclear. AMF and phosphate-solubilizing bacteria originate from different sources, and combining the two is not a natural correlation and may result in an unstable synergistic effect. The mechanism by which compound inoculation with PSMs causes microbial community changes in plant roots is not yet fully understood. Unlike the extensive research on the interactions between microorganisms and plant roots, there is currently limited research on the impact of microbial interactions on plant P-uptake efficiency.

Regarding the research direction of inoculating PSMs to improve the utilization efficiency of plant P, the current focus is to (1) expand the application scope of PSMs, combine functional research such as nitrogen fixation and soil remediation, and increase the application of composite microbial fertilizers to better serve agricultural development. (2) Continuously explore P-solubilization-related genes using a combination of genomics, proteomics, and metabolomics to further explain the mechanisms of soil P dissolution. Single-cell Raman D_2_O technology and high-throughput P-cycling functional gene chips can determine the corresponding functional genes, groups, and activities of PSMs, thereby improving our understanding of the types and functions of PSMs. (3) Strengthening research on microbial interactions and mixed inoculation, which combines AMF with naturally related bacteria, may provide advantages over artificially combined microorganisms. Moreover, by using molecular biology techniques and isotope labeling methods to explore microbial interactions at a deeper level, the optimal effect of microbial interactions in the soil can be achieved, thereby playing an important role in improving soil fertility. (4) Further research is being conducted on the effects of specific chemical substances related to different stages of microbial interactions through a combination of transcriptomics and metabolomics. (5) More attention should be paid to the isolation of indigenous microorganisms, screening for higher-quality and multifunctional PSMs, enhancing plant uptake of soil P, and enhancing their resistance to different stress conditions, which is of great significance for promoting plant growth.

## Author contributions

FP: Writing – original draft, Writing – review & editing. QL: Writing – original draft. MS: Conceptualization, Writing – original draft. ZW: Conceptualization, Funding acquisition, Writing – review & editing. Y-XX: Conceptualization, Writing – review & editing. D-FD: Funding acquisition, Supervision, Writing – review & editing.
